# Identifying Modeled Ship Noise Hotspots for Marine Mammals of Canada's Pacific Region

**DOI:** 10.1371/journal.pone.0089820

**Published:** 2014-03-05

**Authors:** Christine Erbe, Rob Williams, Doug Sandilands, Erin Ashe

**Affiliations:** 1 Centre for Marine Science & Technology, Curtin University, Perth, Western Australia, Australia; 2 Sea Mammal Research Unit, University of St Andrews, St Andrews, Scotland, United Kingdom; 3 Provincetown Center for Coastal Studies, Provincetown, Massachusetts, United States of America; Pacific Northwest National Laboratory, United States of America

## Abstract

The inshore, continental shelf waters of British Columbia (BC), Canada are busy with ship traffic. South coast waters are heavily trafficked by ships using the ports of Vancouver and Seattle. North coast waters are less busy, but expected to get busier based on proposals for container port and liquefied natural gas development and expansion. Abundance estimates and density surface maps are available for 10 commonly seen marine mammals, including northern resident killer whales, fin whales, humpback whales, and other species with at-risk status under Canadian legislation. Ship noise is the dominant anthropogenic contributor to the marine soundscape of BC, and it is chronic. Underwater noise is now being considered in habitat quality assessments in some countries and in marine spatial planning. We modeled the propagation of underwater noise from ships and weighted the received levels by species-specific audiograms. We overlaid the audiogram-weighted maps of ship audibility with animal density maps. The result is a series of so-called “hotspot” maps of ship noise for all 10 marine mammal species, based on cumulative ship noise energy and average distribution in the boreal summer. South coast waters (Juan de Fuca and Haro Straits) are hotspots for all species that use the area, irrespective of their hearing sensitivity, simply due to ubiquitous ship traffic. Secondary hotspots were found on the central and north coasts (Johnstone Strait and the region around Prince Rupert). These maps can identify where anthropogenic noise is predicted to have above-average impact on species-specific habitat, and where mitigation measures may be most effective. This approach can guide effective mitigation without requiring fleet-wide modification in sites where no animals are present or where the area is used by species that are relatively insensitive to ship noise.

## Introduction

The anthropogenic contribution to ocean ambient soundscapes is dominated by commercial shipping in many regions around the world, especially in the northern hemisphere and at low frequencies (10–200 Hz) [1]. In the northeast Pacific, noise from commercial shipping in the inshore waters is the most persistent type of anthropogenic noise, given the large ports of Vancouver and Seattle. In 2008, there were on average three vessels per hour (day and night) in Juan de Fuca and Haro Strait [2]. Unlike many areas of the industrialized northern hemisphere, there has not been any seismic survey exploration in this region for many years. At a local scale, construction-related noise from pile driving or dredging makes ephemeral contributions to the ocean soundscape, but these activities are not considered major sound sources in conservation management plans that address acoustic aspects of this region, and navy exercises using tactical sonars are rare [3], [4].

The potential effects of anthropogenic underwater noise on marine mammals include behavioral responses, communication masking, stress, and—in extreme cases—hearing loss or habitat abandonment [5]–[8]. While regulation of underwater noise in many countries tends to focus on acute noise arising from temporary and impulsive sources (e.g. seismic exploration, pile driving) [9], chronic forms of noise pollution, such as shipping noise, are largely unregulated, although there are indications that European countries may begin to address the issue [10]. In our view, environmental impact assessments have become more holistic in recent years, considering cumulative noise exposure and cumulative stressors [7], [11]–[13]. In many ways, marine mammals have become the icon of the ocean noise issue, possibly because of 1) their popularity with the public, 2) their special protection under the legislation of many countries [14], and 3) evidence that some species may be particularly susceptible to anthropogenic noise [15]. Assessing risk associated with various human activities has been usefully partitioned into *sensitivity* (“the degree to which marine features respond to stresses, which are deviations of environmental conditions beyond the expected range”) and *vulnerability* (“the probability that a feature will be exposed to a stress to which it is sensitive”) [16]. A great deal of research has been done on the sensitivity of marine mammals to noise [6], [17], [18]. Assessing vulnerability involves quantifying overlap in the spatial, temporal and frequency domains.

Assessing the vulnerability of marine mammals to anthropogenic noise has drawn heavily from the scientific literature on ecotoxicology (reviewed in [19]). Much of the scientific attention in this field has focused on quantifying the dose-response relationship between marine mammals and high-amplitude, acute noise sources, especially in cases where dose can lead to adverse behavioral responses that can lead marine mammals to strand [20]. Unfortunately, the dose-response paradigm is proving to be of limited value for quantifying the impacts of chronic forms of ocean noise, which has more in common with habitat degradation or loss than with many other forms of disturbance [5], [11], [21]. Controlled exposure experiments, while informative, are, alone, insufficient to predict consequences of exposing populations of marine mammals to chronic forms of noise. A recurring theme in marine mammal-noise studies is the need to consider the behavioral [22] and ecological context [23]. Very large-scale studies, conducted on the spatial, temporal and spectral scales at which these highly mobile predators live their lives, may be needed to evaluate the influence of changes in a marine mammal's acoustic environment [24]. In recent years, it has become apparent that shipping noise has the potential to mask the opportunities for some low-frequency baleen whales to communicate in highly urbanized waters near ports and shipping lanes [5], [25]. The noise from icebreakers has been shown empirically to mask the communication signals of belugas [26], [27], but logistical constraints will always make it difficult for such experimental studies to be conducted on large baleen whales.

A number of national and international efforts are underway to limit the exposure of marine mammals to chronic forms of ocean noise. At a local scale, many regulators in the UK and Europe are using SAFESIMM to integrate information on marine mammal distribution and the soundfields likely to result from planned noise-generating activities (e.g., seismic surveys or pile-driving) to estimate the number of animals whose dose is likely to exceed given thresholds [28]. On the regulatory side, it appears that the EU has included chronic anthropogenic noise as an indicator of marine habitat quality [10], but quantitative limits are still being debated [29]. In waters under US jurisdiction, the CetSound (http://cetsound.noaa.gov) tools are being developed by NOAA, the US Navy and the Bureau of Ocean Energy Management to compile best available science on soundfield mapping and cetacean distribution. It remains to be seen how these two products, noise map and marine mammal distribution, will be integrated in US management. Canada represents an interesting case study for integrating information on marine mammals and noise. A legal challenge has upheld Canada's obligation to protect acoustic elements of critical habitat for resident killer whales, but there is little guidance in the scientific literature on how to do so for killer whales, and it is unclear whether Canada will include sound as a primary constituent element of critical habitat for other acoustically sensitive species under the Species at Risk Act [3], [30].

Global patterns in ship traffic are so firmly entrenched [31] that the problem of chronic ocean noise impacts on marine mammals is best viewed in spatial terms. By framing the issue in those terms, it becomes possible to draw on a wealth of experience in natural resource management in terms of spatial planning tools to separate vulnerable species from threatening processes [32], [33]. The motivation for our study was to integrate best available science from the continental shelf waters of Canada's Pacific region on both animal distribution patterns and ship noise. Simply being in the same place and time as noise does not mean that an animal will be affected by noise, but spatial and temporal overlap with noise is a necessary precursor to risk [34]. In the spectral domain, it is important to note that marine mammals are a diverse guild of predators with a diverse range of hearing abilities. Anthropogenic noise is perceived in quite different ways by marine mammals with different auditory systems. From a practical, marine mammal conservation and management standpoint, it may be a lower priority to reduce noise levels in places that are not used by marine mammals capable of hearing low-frequency sound than in places that are of critical importance to species whose hearing is most sensitive in low frequencies. We say “may be”, because indirect effects of noise can be mediated by effects on prey or predators, which is beyond the scope of this study. We illustrate some key elements of a spatially explicit risk assessment [35] for marine mammals and noise in the northeast Pacific, by integrating information on average distribution and abundance of 10 marine mammal populations, cumulative acoustic energy from ships, and our best estimate of how that acoustic energy may be perceived by the auditory system of the various marine mammals in question.

## Methods

### Marine Mammal Density Maps

**Figure 1:**
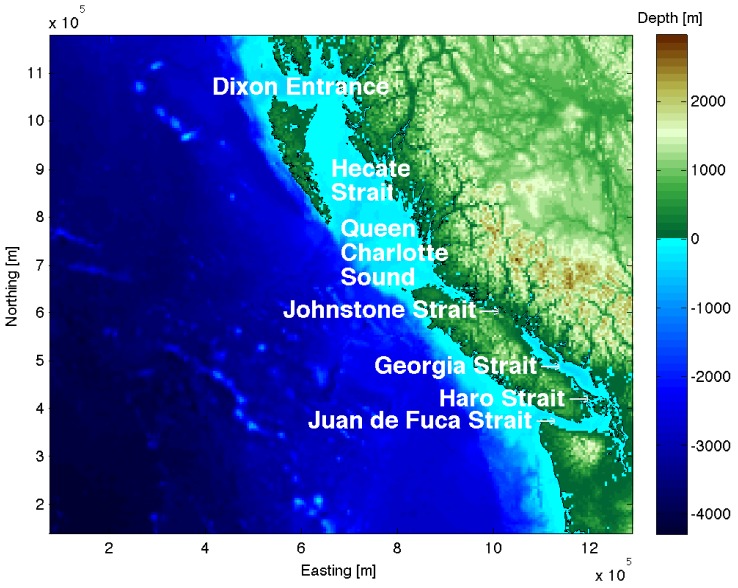
Map of British Columbia inshore waters identifying major waterways.

Systematic line transect surveys were conducted in summer months 2004–2005, resulting in abundance estimates for 6 cetacean (whale, dolphin and porpoise) and one pinniped (seal or sea lion) species in British Columbia (BC, see Fig. 1) waters [36]. An additional season of survey effort and the use of advanced, spatial modeling techniques generated interpolated density maps that showed average distribution of 11 marine mammal species from these surveys [37]. These include harbor seal (*Phoca vitulina*) and elephant seal *(Mirounga angustirostris)*; Steller sea lion (*Eumetopias jubatus*); Dall's (*Phocoenoides dalli*) and harbor porpoise (*Phocoena phocoena*); fin (*Balaenoptera physalus*), common minke (*Balaenoptera acutorostrata*), humpback (*Megaptera novaeangliae*) and northern resident killer whales (*Orcinus orca*); Pacific white-sided dolphin (*Lagenorhynchus obliquidens*); and sea otter (*Enhydra lutris*). Collectively, these density surface maps provide a snapshot of the typical summertime distribution of 11 marine mammal populations along the continental shelf of Canada's Pacific waters. Although some of these species may be studied using methods other than line transect surveys (e.g., photo-identification), our spatial conservation prioritization process requires distribution to be approximated by a surface, rather than point data. Density surface models were used in study, to be consistent with a previous spatially explicit ship strike risk assessment [38] and biodiversity assessment [39], but the response variable could be any continuous variable that can be plotted as a surface (e.g., probability of occurrence, frequency of occurrence, relative environmental suitability, occupancy).

### Marine Mammal Audiograms

**Figure 2:**
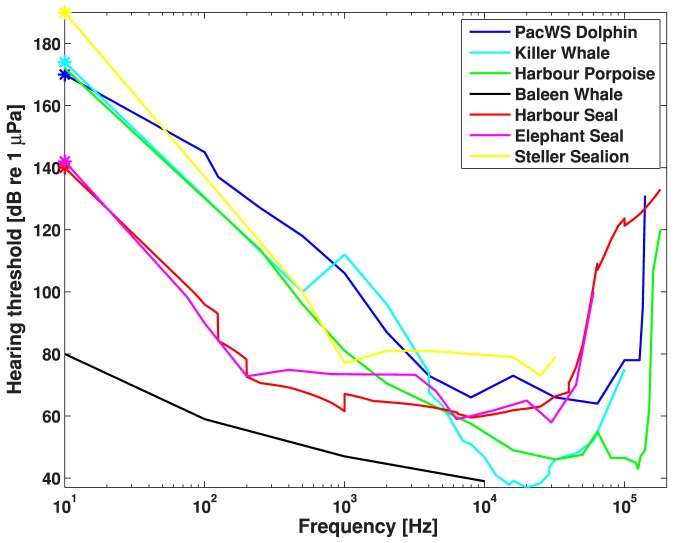
Audiograms of marine mammal species occurring in British Columbia.

Audiogram data for Pacific white-sided dolphins were taken from the literature [40]. For harbor porpoises, we used the mean of [41] and [42] at long signal durations. This audiogram was also applied to Dall's porpoise, because direct measurements do not exist for Dall's porpoise. For killer whales, we took the mean of two published behavioral audiograms [43], [44], noting that the low-frequency thresholds in the latter article might have been noise-limited. In the absence of audiograms for baleen whales, we followed the recommendation of [45], and used the lower envelope of natural ambient noise [46], and raised this by 20 dB to estimate hearing sensitivity in fin, humpback and minke whales. This is based on the assumption that the frequency band of best hearing sensitivity includes the frequencies of sound production, and that animal hearing evolved such that the sensitivity would be above persistent ambient noise levels, in order to make maximum use of the dynamic range of the animal's auditory system [45]. In the absence of critical ratio data for baleen whales, a 20 dB critical ratio typical for other marine mammals at mid-to-low frequencies <10 kHz [17], [47] was applied. We took the published audiogram for elephant seals [48], [49] and the mean of all published audiograms for harbor seals [48], [50]–[53]. For Steller sea lions, we used the minimum of the male and female audiograms, as the female was significantly more sensitive (>15 dB) than the male [54]. An audiogram was not available for sea otters, so we ignored this species in subsequent analyses, bringing the number of species we assessed from 11 to 10. We extrapolated all audiograms down to 10 Hz by extending the slope over the three lowest-frequency measurements. Fig. 2 shows the audiograms used to estimate the audibility of ship noise.

### Vessel Data

**Figure 3:**
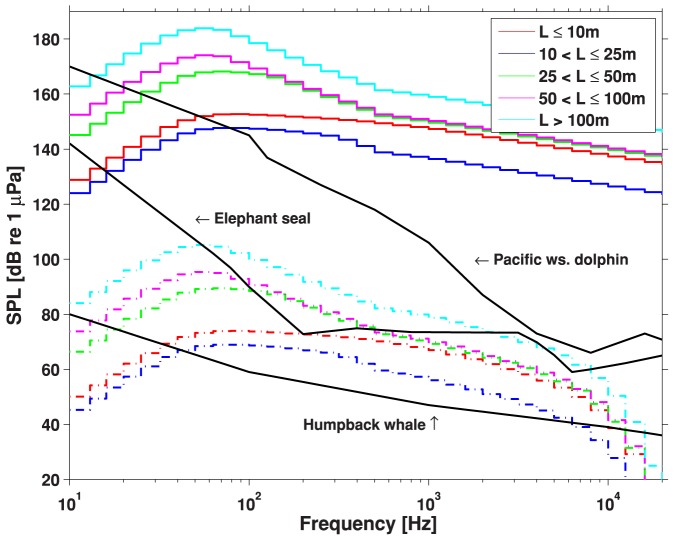
Ship source spectra (1/3 octave band levels) for five vessel length classes (solid lines) in dB re 1 µPa @ 1 m. Note that vessels <10 m length were louder than vessels up to 25 m length due to the faster mean speed of the shorter vessels. Received spectra at 30 km range in 50 m of water (dash-dot lines), in dB re 1 µPa. Marine mammal audiograms from Fig. 2 (black lines) in dB re 1 µPa.

Ship locations were determined from Automatic Identification System (AIS) data logged by the Canadian Coast Guard. The total time spent in each cell of a 5 km×5 km grid was computed for five vessel classes over the period June - September 2008 [2], which corresponds to the months (but not year) during which the marine mammal survey data were collected. Ship source spectra were estimated by the Research Ambient Noise Directionality (RANDI) model [55] as a function of vessel length (directly available in the AIS data) and mean speed (computed over successive AIS logs). Vessels were grouped into five length classes [2]. Vessel source spectra modeled in RANDI were extended to 20 kHz based on a decrease in power spectrum density of 20 dB per decade in frequency [56]. This is equal to a decrease of 10 dB per decade in frequency for 1/3 octave band levels. The resulting 1/3 octave source levels are shown as solid lines in Fig. 3.

### Audibility of Ships

As ship noise propagates away from the source, the band levels decrease relative to the animal audiogram. Volumetric absorption due to the molecular relaxation of seawater is stronger at higher frequencies than at lower frequencies. As a conceptual example, Fig. 3 shows the received ship noise spectra at 30 km range in 50 m deep water as dash-dot lines. For comparison, the audiograms of Pacific white-sided dolphins, elephant seals and humpback whales are plotted as well. Only energy above the audiogram is assumed audible in the following audibility assessment. In the above example, even the loudest ships are no longer audible to Pacific white-sided dolphins at 30 km range and beyond. Energy around 200 Hz remains audible over the longest ranges in the case of elephant seals. For baleen whales, the low-frequency peak of the ship spectrum at 50 Hz remains audible over the longest ranges. This plot illustrates how the audiogram weighting determines which frequencies will be audible over the farthest ranges, and how these spectral characteristics differ amongst species.

Received levels of ship noise were computed on a 5 km×5 km grid over a 100 km radius from each source cell (i.e. each cell with ship logs). To propagate ship noise through the marine environment, a geometric spreading model was applied decreasing the noise level by 20 log10(range/m) until the range equaled the maximum water depth along the specific source-cell to receiver-cell transect, and by 10 log10(range/m) thereafter. Bathymetry was obtained from the Canadian Hydrographic Service. Frequency-dependent, volumetric absorption was also accounted for [57], [58], and results in the faster loss of energy at higher frequencies than at lower frequencies. The received level in each source cell was computed via the propagation loss over 2 km range, which is the average distance from the center of a 5 km×5 km cell. Hence the noise map does not show any source levels; even in source cells, the level plotted is the sum of all contributions from neighboring cells plus the contribution from ships within this cell propagated over 2 km. Up to this point, the methods have been described in more detail elsewhere [2].

The ship spectrum received at each receiver cell was filtered by the animal audiogram. The audible energy in each receiver cell was integrated over all ship positions within 100 km radius, over all vessel classes, over frequency and over time. The result was a map representing audible acoustic energy from shipping over the summer (June-September) of 2008 for each species.

### Cumulative Ship Noise

As a first-order validation exercise, we compared our predicted cumulative sound exposure levels without audiogram-filtering to measured underwater noise reported recently for 12 sites in our study area [59]. The modeled unweighted cumulative noise from shipping over the year of 2008 was read off Fig. 2a [2] at each of the 12 empirical sampling locations. The modeled cumulative sound exposure levels were ranked from noisiest (1) to quietest (12). The median noise level measured at each site over 20–166 days between 2008 and 2010 was extracted from Table S1 in reference [59] in each of three frequency bands (17–28 Hz; 71–708 Hz; and 1500–3500 Hz), and also ranked from 1–12. A Spearman rank-order correlation (i.e., the nonparametric version of the Pearson correlation) was used to measure the strength of association between the two ranked variables, namely predicted cumulative sound exposure level [2] and recorded ambient noise level [59].

### Noise-Density Hotspots

The audibility maps were limited to the area that had previously been surveyed for marine mammals [36], [37], i.e. the BC continental shelf waters. The audibility maps were scaled to range from 0 to 1 by subtracting the minimum received energy over all cells from the entire map, and by dividing the audibility map by the maximum received energy. This was done for each species. The density maps were normalized to 0–1 the same way. The normalized noise audibility map and the normalized density map were multiplied for each species. In areas where the audible energy was high (i.e. close to 1) and where animal density was high (i.e. close to 1) the product was high, indicating a “hotspot”. In areas where either the audible energy or the animal density was high and the other one was low, the product was low (i.e. close to zero) indicating a region of little concern. The product of the two maps was normalized to 0–1 as well, to yield a risk index for each species. Risk indices computed this way are not comparable among species (as the map for each species was normalized to 0–1), but can be used to rank habitat for each species.

## Results

### Cumulative Ship Noise

There was a significant correlation between the rankings, from noisy to quiet, of the modeled noise levels and the empirical measurements of underwater noise. In the lowest frequency band corresponding to fin whale calls (17–28 Hz), the Spearman's rank correlation coefficient (r_s_) was 0.7295 (t = 3.02; df = 8; two-tailed P = 0.017). In the 71–708 Hz band, r_s_ was 0.8815 (t = 5.28; df = 8; two-tailed P = 0.0007). In the 1500–3500 Hz band, r_s_ was 0.8937 (t = 4.13; df = 10; two-tailed P = 0.0020). Note that noise statistics were available for 12 sites in the highest frequency band, but only 10 in the lower and mid- frequency bands [39]. We are therefore confident that our predicted noise surface provides a reliable proxy for scoring habitat from noisy to quiet sites.

### Audibility of Ships

**Figure 4:**
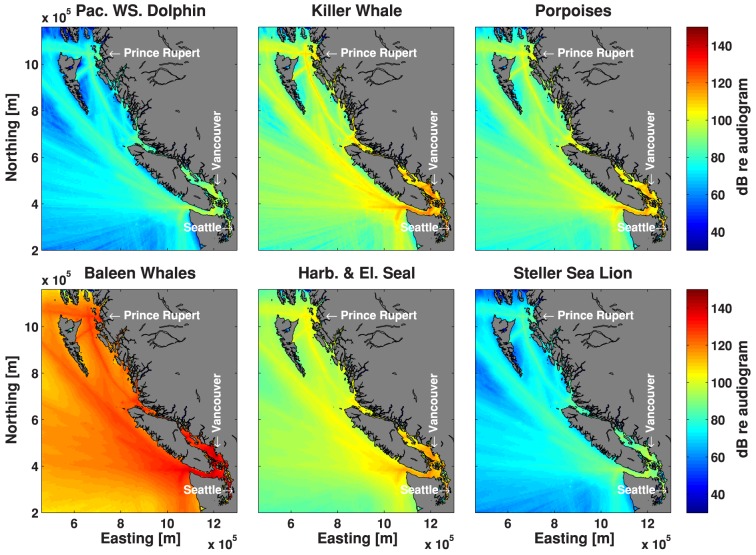
A measure of audible acoustic energy from all classes of ships over the summer of 2008 by species on a 5×5 km grid.

A measure of total acoustic energy from all ships over the summer of 2008 is shown for six of the seven audiograms in Fig. 4. Given the similarity of the elephant and harbor seal audiograms, only the latter is shown. Animals with the least hearing sensitivity below 20 kHz (Steller sea lions and Pacific white-sided dolphins) are expected to perceive the least amount of acoustic energy. Animals with better hearing sensitivity at low-to-mid frequencies (50–300 Hz) experience the most ship noise (baleen whales and true (phocid) seals).

### Noise-Density Hotspots

**Figure 5:**
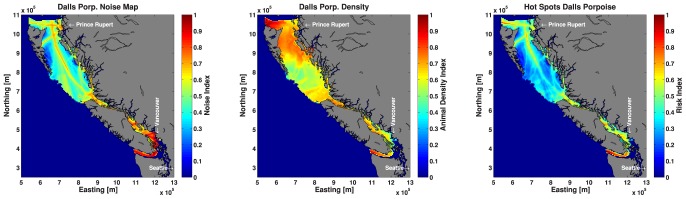
Audibility map for Dall's porpoise (left), Dall's porpoise density map (middle) and resulting map of hotspots (right).

**Figure 6:**
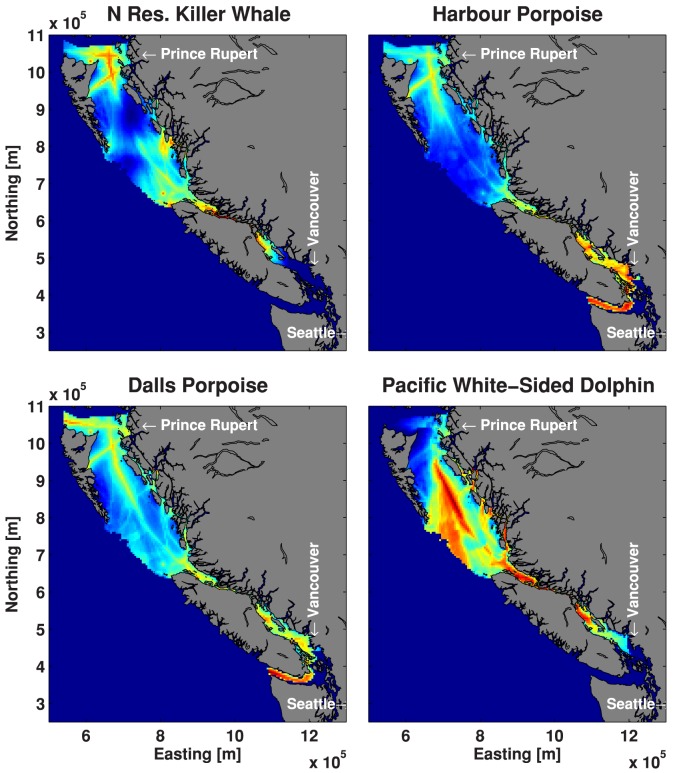
Map of ship noise and animal hotspots for four odontocete species.

**Figure 7:**
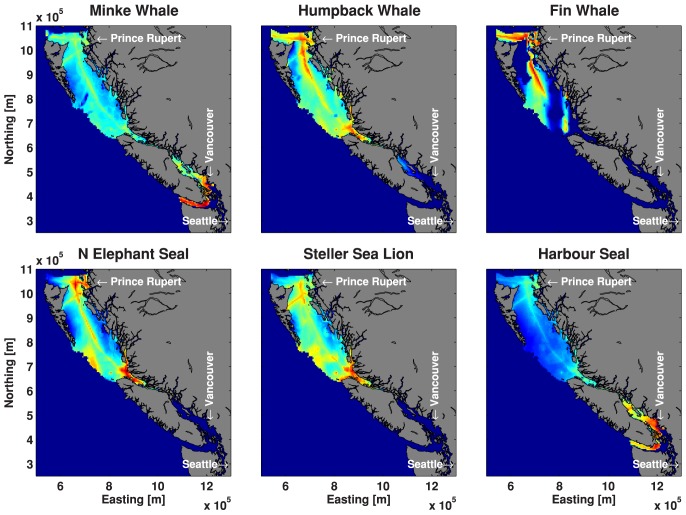
Map of ship noise and animal hotspots for baleen whales and pinnipeds. All color scales range from 0 (blue) to 1 (red), indicating least to highest risk.

To illustrate the process we used to map risk (i.e., vulnerability and sensitivity), Fig. 5 shows the audibility map for Dall's porpoise normalized to 0–1, the Dall's porpoise density map normalized to 0–1 and the product of these two maps normalized to 0–1, i.e. the map of hotspots. In terms of results, Fig. 6 shows the hotspots for all of the odontocete species, and Fig. 7 shows the maps of hotspots for baleen whales and pinnipeds occurring in BC waters.

For species that exist in Juan de Fuca and Haro Strait, these regions are hotspots due to the large amount of ship traffic and hence ship noise. For populations that do not range this far south, Johnstone Strait and the waters off Prince Rupert tend to have the highest risk index. Secondary hotspots were identified somewhat removed from the major shipping lanes, e.g. near shore and in fjord entrances where coastal seals and dolphins are common. Ship noise does not propagate well into the narrow and winding fjords, which represent important habitats to some species; however, with increasing onshore development, ship noise in fjords is likely to increase.

## Discussion

We compared modeled ship noise in British Columbia with measured and modeled animal density data. The geometric sound propagation model ignored spatiotemporal differences in the acoustic environment of the water column and the seafloor. The spatiotemporal variability of the sound propagation model and its uncertainty were discussed elsewhere [2]. 2008 was a year of the global economic downturn, which might have resulted in reduced shipping activity, specifically of large cargo-vessels. Shipping routes did not change, and as the noise maps are normalized, we expect the geographic hotspots to be unaffected.

In the absence of population- and situation-specific data on noise impacts, noise mitigation in the real world tends to involve the application of simple “do-not-exceed” thresholds, for broad taxonomic groups such as pinnipeds, mysticetes and odontocetes [7], [28]. Our approach differentiates among species by applying an audiogram-weighted metric corresponding to our best estimate of received acoustic energy. As a result, the geographic areas and the extent of the areas in which ship noise might impact marine mammals differ from species to species. Hearing sensitivity varies amongst individuals of the same species [60]; the audiograms of 14 (3–15 year-old) bottlenose dolphins varied by up to 10 dB [61]. Hearing loss with age (>20 years) has been shown in bottlenose dolphins (*Tursiops truncatus*), progressing from high to low frequencies, and being worse in males than females [62], [63]. Life history and sound exposure history of captive animals, whose audiograms have been measured, are often unknown. We also note the scarcity of hearing data for some species, with the Pacific white-sided dolphin and elephant seal audiograms being based on one animal. M-weighting has been recommended to group marine mammal species into functional groups for bioacoustic impact assessments of strong sounds [7]. For the assessment of lower-level responses such as behavioral changes and masking, audiogram weighting has been preferred [64].

Correlating the resulting ship-audibility maps with density surface maps for each species yielded patterns of hotspots for each species. In other words, the same noise surface carries quite different consequences for conservation and management of different marine mammal species, because different distribution patterns cause the species to differ widely in their vulnerability (exposure to noise), whereas different hearing abilities cause the species to differ widely in their sensitivity (in this case, ability to perceive anthropogenic noise). We suggest this audiogram-weighted approach for chronic ship noise as a means of differentiating between species based on the received acoustic energy, which might correlate with audibility-dependent impacts such as behavioral responses or masking. We do not advocate this approach for impact assessments of acute, intense exposures as from seismic surveying or pile driving. Based solely on the physical properties of sound in the ocean, we postulate that marine mammals that hear best in the frequency bands dominated by ship noise should be most affected by high levels of ship noise, but this may not be true. It is conceivable that natural selection is particularly active at the edge of audibility (where the acoustic arms-race between predator and prey is taking place [65]), and that the ability to detect signals in noise at the edge of audibility is a key determinant of survival and reproduction. We consider the various ways of weighting received level by hearing sensitivity as hypotheses to be tested with new behavioral response data. Our maps showing areas where these different species are and are not currently experiencing high levels of anthropogenic noise would be useful in choosing experimental control and treatment sites for future experiments to understand the responsiveness of different species to noise.

These risk maps can inform marine spatial planning efforts, but they are only one input into a systematic conservation planning process [32]. Future tasks require managers and policy makers to set explicit conservation targets, which may vary according to the conservation status of each population. Future iterations could incorporate additional data, as long as other datasets can be modeled to account for spatial bias in opportunistic sightings, photo-ID locations, or data from non-randomized surveys. Otherwise, managers may end up inadvertently protecting sites where it is convenient to collect data, rather than sites that are most important to at-risk species. The percentage of any species' habitat that is affected by noise can be read off a map. The areas where noise is high and animal density is high can also be identified in our risk maps, indicating where marine spatial planning efforts have the most impact. It is worth noting that the critical habitats for northern resident killer whales in Johnstone Strait, for example, are quite noisy (Figure 6), although there is a legal obligation in Canada to manage acoustic elements of the critical habitats of these whales [3]. In contrast, species that rely on Hecate Strait waters (e.g., humpback and fin whales, and Pacific white-sided dolphins) enjoy relatively quiet waters, although we are unaware of any legal obligation to keep these waters quiet.

Although we have outlined one defensible way to combine information on chronic ocean noise and marine mammal habitat use, there are a number of technical issues for us to resolve before these predictions are ready for use in real-world management. First, many sound sources are simply missing from this estimate of cumulative ship noise energy. The most important of these missing sources in our noise maps is small boat traffic. Small boats do not log AIS positions, and can exist in large numbers in certain areas for recreational fishing, boating or whalewatching [66], [67]. Repeated disturbance from small boats can disrupt feeding in killer whales [22] and alter the behavior of humpback whales [68]. Secondly, additional efforts are needed to validate these predictions in absolute rather than relative terms (i.e., ranking) with empirical data. Thirdly, noise mapping and animal surveys were correlated for the months of June-September, when the surveys had been carried out. Noise exposure during the rest of the year has not been included.

It should be noted that, although we have used density surface models as a convenient way to illustrate the average distribution of the species in our study, there are many other ways to report distribution and habitat use. Like any spatial conservation prioritization exercise, our methods require information that can be used to assign priorities to different sites. In practice, this means that for a gridded study area like ours, one needs a value for each cell and those values need to be in a common currency. In our example, we have used a predicted value of density, which relies on well-established statistical methods. The methods would also work with information on probability or frequency of occurrence, relative habitat suitability, or any robust measure of relative abundance. Many cetacean studies collect “point” data, in the process of collecting photo-identification or biopsy data. It would be important to use appropriate statistical models to account for any spatial bias in such data, to avoid the situation in which an area that is a convenient place to collect data becomes mistaken for a high-priority area to protect [39].

It is hoped that our efforts could serve three purposes: (a) as a current best estimate of co-occurrence of marine mammals and chronic ocean noise levels in Canada's Pacific region, to add to the “best available science” base as Canada sets priority species and regions for conservation, management and mitigation; (b) as a framework for making predictions about the consequences likely to result from increased noise levels as various parts of the coast are subject to industrial development applications, or conversely as places where ship-quieting technologies may be most useful; and (c) as a simple audiogram-weighting method that could be used anywhere that a variety of marine mammal species may be at risk from chronic anthropogenic noise.
