# Correction: Identifying Modeled Ship Noise Hotspots for Marine Mammals of Canada's Pacific Region

**DOI:** 10.1371/journal.pone.0114362

**Published:** 2014-11-26

**Authors:** The PLOS ONE Staff

The following information is missing from the Acknowledgments section: We thank Patrick O’Hara and his colleagues in the Oil-in-Canadian-Waters research group (Rosaline Canessa, Ron Pelot and Norma Serra) for extensive analysis of vessel count, speed and location data to facilitate our acoustic analyses.

There are several errors in the References section. References 3, 4, 28, 29, 30, 36, 37, 38, 39, and 55 are incorrect. The correct references are as follows:

3. Fisheries and Oceans Canada (2011) Recovery Strategy for the Northern and Southern Resident Killer Whales (Orcinus orca) in Canada. Species at Risk Act Recovery Strategy Series. Ottawa: Fisheries and Oceans Canada.

4. National Marine Fisheries Service (2008) Recovery Plan for Southern Resident Killer Whales (Orcinus orca). Seattle, WA, USA: National Marine Fisheries Service, Northwest Region. 251 p.

28. Donovan CR, Harris C, Harwood J, Milazzo L. A Simulation-Based Method for Quantifying and Mitigating the Effects of Anthropogenic Sound on Marine Mammals; 2012; Edinburgh, Scotland. Proceedings of Meetings on Acoustics.

29. Federal Court (2010) Northern and Southern Resident Killer Whales (Orcinus orca) in Canada: Critical Habitat Protection Statement. In: Ecojustice, editor. 2010 FC 1233 Ottawa, Ontario, Canada: Federal Court of Appeal. pp. 127.

30. Fisheries and Oceans Canada (2010) Recovery Strategy for the North Pacific Humpback Whale (Megaptera novaeangliae) in Canada [DRAFT]. In: Canada FaO, editor. Ottawa: Government of Canada. pp. x + 51 pp.

36. Williams R, Thomas L (2007) Distribution and abundance of marine mammals in the coastal waters of British Columbia, Canada. Journal of Cetacean Research and Management 9: 15-28.

37. Williams R, Ashe E, O'Hara PD (2011) Marine mammals and debris in coastal waters of British Columbia, Canada. Marine Pollution Bulletin 62: 1303-1316.

38. Williams R, O'Hara P (2010) Modelling ship strike risk to fin, humpback and killer whales in British Columbia, Canada. Journal of Cetacean Research and Management 11: 1-8.

39. Williams R, Grand J, Hooker SK, Buckland ST, Reeves RR, et al. (2014) Prioritizing global marine mammal habitats using density maps in place of range maps. Ecography 37: 212–220.

55. Breeding JE, Pflug LA, Bradley M, Walrod MH, McBride W (1996) Research Ambient Noise Directionality (RANDI) 3.1 Physics Description. Planning System Incorporated.
